# Discordant twins with the smaller baby appropriate for gestational age – unusual manifestation of superfoetation: A case report

**DOI:** 10.1186/1471-2431-7-2

**Published:** 2007-01-19

**Authors:** Noopur Baijal, Mohit Sahni, Neeraj Verma, Amit Kumar, Nittin Parkhe, Jacob M Puliyel

**Affiliations:** 1 Department of Pediatrics and Neonatology St Stephens Hospital, Tis Hazari, Delhi 110054, India; 2 Department of Radiology, St Stephens Hospital, Tis Hazari, Delhi 110054, India

## Abstract

**Background:**

Documentation of superfoetation is extremely rare in humans., The younger foetus has invariably been small for gestational age (estimated from the date of the last menstrual bleed) in all the cases reported in the literature. We report a case where the younger twin was of appropriate size for gestation.

**Case Presentation:**

The first of twins was of 32 weeks gestation and the baby was of appropriate size and development for the gestational age. The second twin was of 36 weeks gestation. Gestational age was estimated with the New Ballard score, x-ray of the lower limbs, dental age on x-ray, and ophthalmic examination.

**Conclusion:**

Bleeding on implantation of the first foetus probably helped demarcate the two pregnancies. Dental age and the New Ballard score can be used to diagnose superfoetation in discordant twins, when detailed first trimester ultra-sound data is not available.

## Background

Superfoetation implies fertilization and subsequent development of an ovum when a foetus is already present in the uterus. Growth discordance in multiple pregnancies due to placental insufficiency, twin to twin transfusion or aneuploidy need to be differentiated from superfoetation. In most instances the larger twin is nearer appropriate size for gestational age calculated from the last menstrual period (LMP). We report a case where superfoetation was likely superfoetationbecause the smaller of the twin was of appropriate maturity, weight and length for gestational age. These circumstances argued against intrauterine growth retardation in the smaller twin.

## Case Presentation

A 21-year-old mother of two had an ante-natal ultrasound examination done 26 weeks after her last menstrual period (LMP). This showed twins, one was of appropriate size for duration of amenorrhoea while the other was approximately four weeks too large. The ultrasound findings are described in Table 1.

**Figure 1:**
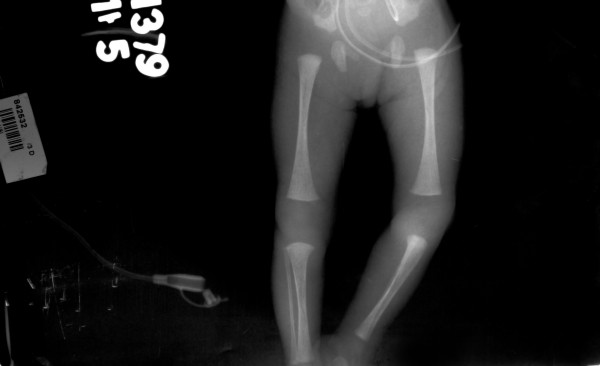
Radiological examination for bone showing absence of epiphysis at the lower end of femur and upper end of tibia in Twin A.

**Figure 2:**
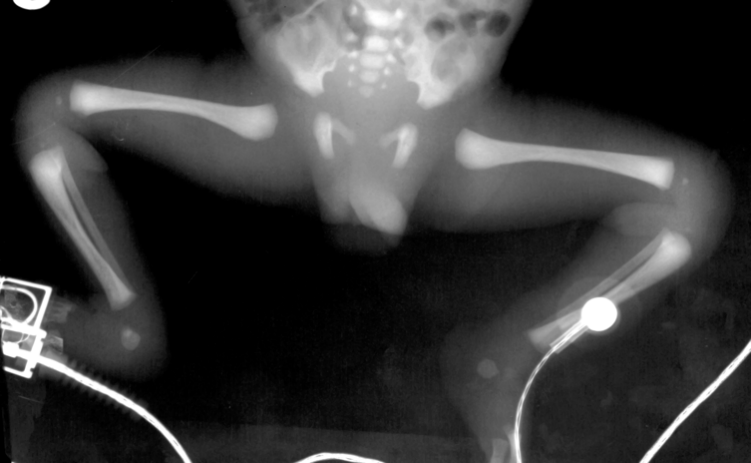
Radiological examination for bone showing epiphysis at the lower end of femur and upper end of tibia in Twin B.

Six weeks later, after 32 weeks of amenorrhoea, live twins were delivered. They had not received ante-natal steroids. The first of the twins (Twin A) weighed 980 grams and the next baby (Twin B) weighed 2160 grams. Detailed neurological assessment using the New Ballard Scoring [1], was done on the second day. The score for Twin A was 15, pointing to a gestational age of 30 weeks (+/- 2 weeks) and the score for Twin B was 32, appropriate for 36 weeks (+/- 2 weeks). Table 2 lists the differences between the twins. Radiological examination for bone age done on the second day of life revealed the absence of epiphysis at the lower end of femur and upper end of tibia in Twin A while they were present in Twin B (Figure 1, 2). The epiphysis at the lower end of femur appears normally between 31 and 40 weeks and for the upper end of tibia between 34 weeks and 5 postnatal weeks [2]. Thus, Twin A had a bone age of less than 31 weeks and Twin B had a bone age of at least 34 weeks.

**Figure 3:**
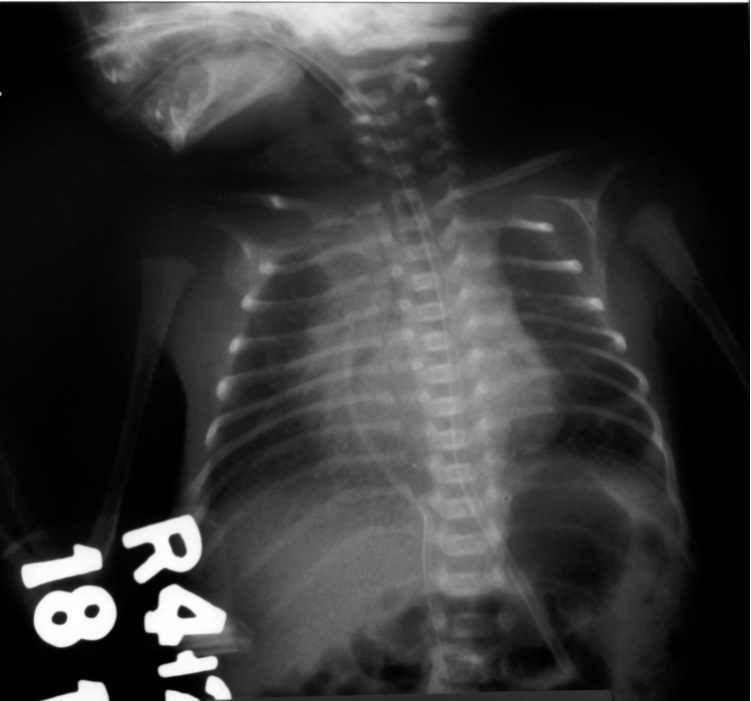
X-ray chest with mandible showed absence of calcified crowns of the first and second deciduous molar in Twin A.

**Figure 4:**
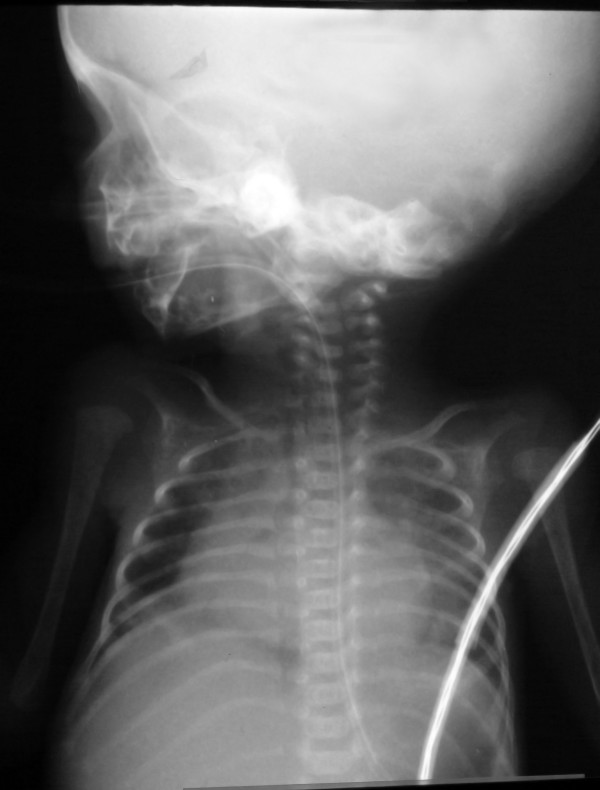
X-ray chest with mandible showed calcified crowns of the first and second deciduous molar in Twin B.

X-ray chest with mandible showed absence of calcified crowns of the first and second deciduous molar in Twin A and both crowns calcified in Twin B (Figure 3, 4). The crowns of the first and second molars are never seen prior to 33 and 36 weeks respectively and are invariably seen after that [3]. This suggests that twin A was at less 33 weeks and twin B was at least 36 weeks old.

Retinal vessels normally reach nasal ora serrata by 36 weeks and periphery on the temporal side by 40 weeks.

Ophthalmological examination of Twin Ashowed a hazy cornea and the underlying papillary membrane was not visualised. The retinal vessels had not reached the nasal ora serrata. In Twin B the cornea was clear, there was no papillary membrane and the retinal vessels migration was complete on the nasal side and near complete on the temporal side.

## Conclusion

Intrauterine growth retardation (IUGR) is the usual cause of discordance in multiple pregnancies. We did not find anyreport in the literature of discordance due to one baby being large-for-date. In this case the smaller twin was of appropriate size and maturity for gestation assessed from LMP. The second twin was approximately a month too large and mature. Superfoetation was considered as a possible explanation for the observation. Bleeding one month after conception occurs in about 8% pregnancies and represents a physiological response to implantation or slight bleed from the endometrium in early pregnancy [4]. We therefore also considered the possibility that both twins were conceived simultaneously a month prior to the presumed date of the LMP, and the smaller Twin A was small-for-date.

Detailed neurological and physical assessment is considered the most reliable method of estimation of gestational age, in circumstances where IUGR is suspected and there is uncertainty in using LMP [2]. Using the New Ballard Score [1] the first of the twin was 30 weeks and the second was 36 weeks (+/- 2 weeks). This evidence of disparity in the gestational ages of the 'twins', was corroborated by the estimation of age based on anthropometric measurements, weight, length and head circumference, ophthalmic examination, bone age and dental age estimates. The twins had not received ante-natal steroids which, which had they been given, may have influenced some of the markers of foetal maturity. The evidence taken together, suggests that there was a real difference of approximately 4 weeks in the gestational ages of the twins and this was in keeping with the findings of the ante-natal ultrasound examination.

Among the evidence listed above, anthropometric measurements and bone maturation are delayed in first-trimester-malnutrition and results in symmetric growth retardation [2,5]. However the work of Kuhns et al [3] suggest that the age of calcification of the crowns of the molars is not affected by IUGR and we use this criterion along with the New Ballard Score and the ophthalmic examination to confirm the disparity in gestational ages of the neonates. Harrison et al [6] have recently reported a case of superfoetation and suggested that in growth-discrepant multiple deliveries, skilled neurosonography and ophthalmic examination may be used to support the diagnosis of superfoetation when detailed first trimester data is lacking. We would like to add the role of the New Ballard Scoring and, as they may help clinch the diagnosis, even where suggestive fortuitous circumstances as in this case (distinct marker separating the two fertilizations in the form of bleeding on implantation of first ovum) are not available.

## Competing interests

The author(s) declare that they have no competing interests.

## Authors' contributions

NB, SM, NV and AK investigated the case, searched the literature and helped with the write up, NP helped with the radiological input, and JMP conceived of the study and helped with its write up. All authors read and approved the final manuscript.

## Pre-publication history

The pre-publication history for this paper can be accessed here:



## Figures and Tables

**Table 1:** Ante-natal ultrasonography findings at 26 weeks after LMP

	**Twin A**	**Twin B**
Presentation	Cephalic	Breech
Placenta	Anterior	Posterior
Biparital diameter	65 mm	77 mm
Femur Length	49 mm	48 mm
Head circumference	235 mm	276 mm
**Gestation**	**26 weeks +/- 2 weeks**	**30 weeks +/- 2 weeks**

**Table 2:** Differences between the twins at birth

	Twin A (Gestational age range in weeks +/-2SD)	Twin B (Gestational age range in weeks +/-2SD)
Weight (gm)*	980 (26–30)	2160 (30–36)
Length (cm)*	36.5 (27–29)	42.5 (30–34)
Circumference head (cm)*	27 (27–31)	31 (31–37)
New Ballard Score	15 (30 +/- 2 weeks)	32 (36 +/- 2 weeks)
Epiphysis lower end of femur	Absent (Less than 31 weeks)	Present (31 – 40 weeks)
Epiphysis upper end tibia	Absent (Less than 34 weeks)	Present (34 – 5 post natal weeks)
Calcified crown of 1^st ^deciduous molar	Absent (less than 33 weeks)	Present (more than 33 weeks)
Calcified crown of 1^st ^deciduous molar	Absent (less than 36 weeks)	Present (more than 36 weeks)
Cornea	Haze present	Transparent
Retinal vessels migration	Not reached nasal ora serrata (less than 36 weeks)**	Complete on the nasal side and near complete on the temporal side. (more than 36 weeks)**

*Norms for anthropometry: Usher R et al J Ped 1969 74 901

**Ophthalmological norms: Moller HU. Milestones and normative data. *In: *Taylor D. editor Pediatric Ophthalmology 2^nd ^en. Blackwell Science1997; pp 42–54.

References for other norms are described in the text
